# ULK1 inhibition as a targeted therapeutic strategy for FLT3-ITD-mutated acute myeloid leukemia

**DOI:** 10.1186/s13046-020-01580-4

**Published:** 2020-05-11

**Authors:** Doh Yu Hwang, Ju-In Eom, Ji Eun Jang, Hoi-Kyung Jeung, Haerim Chung, Jin Seok Kim, June-Won Cheong, Yoo Hong Min

**Affiliations:** 1 Division of Hematology, Department of Internal Medicine, Severance Hospital, Yonsei University College of Medicine, 50-1 Yonsei-ro, Seodaemun-gu, Seoul, 03722 South Korea; 2 Avison Biomedical Research Center, Yonsei University College of Medicine, Seoul, 03722 South Korea

**Keywords:** FLT3-ITD mutation, Acute myeloid leukemia, ULK1, Apoptosis, Autophagy

## Abstract

**Background:**

In acute myeloid leukemia (AML), internal tandem duplication mutations in the FLT3 tyrosine kinase receptor (FLT3-ITD) are associated with a dismal outcome. Although uncoordinated 51-like kinase 1 (ULK1), which plays a central role in the autophagy pathway, has emerged as a novel therapeutic target for various cancers, its role in FLT3-ITD AML remains elusive. In this study, we evaluated the effects of ULK1 inhibition on leukemia cell death in FLT3-ITD AML.

**Method:**

We evaluated ULK1 expression and the levels of apoptosis and autophagy following ULK1 inhibition in FLT3-ITD AML cell lines and investigated the mechanism underlying apoptosis induced by ULK1 inhibition. Statistical analysis was performed using GraphPad Prism 4.0 (GraphPad Software Inc).

**Results:**

FLT3-ITD AML cells showed significantly higher ULK1 expression than FLT3-wild-type (WT) AML cells. Two ULK1 inhibitors, MRT 68921 and SBI-0206965, induced apoptosis in FLT3-ITD AML cells, with relatively minimal effects on FLT3-WT AML cells and normal CD34-positive cells. Apoptosis induction by ULK1 inhibition was associated with caspase pathway activation. Interestingly, ULK1 inhibition paradoxically also induced autophagy, showing synergistic interaction with autophagy inhibitors. Hence, autophagy may act as a prosurvival mechanism in FLT3-ITD AML cells. FLT3-ITD protein degradation and inhibition of the ERK, AKT, and STAT5 pathways were also observed in FLT3-ITD AML cells following treatment with ULK1 inhibitors.

**Conclusion:**

ULK1 is a viable drug target and ULK1 inhibition may represent a promising therapeutic strategy against FLT3-ITD AML.

## Background

FMS-like tyrosine kinase 3 internal tandem duplication (FLT3-ITD)-mutated acute myeloid leukemia (AML) accounts for up to 30% of adult AML cases, and the prognosis of patients with FLT3-ITD-mutated AML is extremely dismal owing to the high relapse rate after chemotherapy and allogeneic stem cell transplantation [1]. Activating ITD mutations in *FLT3* increases cell proliferation and survival, while blocking cellular differentiation through the constitutive activation of canonical pathways such as MAPK/ERK, PI3K/AKT, and STAT5; these mechanisms, together with other recurrent molecular abnormalities, are implicated in AML induction [2].

Several FLT3 tyrosine kinase inhibitors (TKIs) have been developed to target the aberrantly activated FLT3 receptor and to suppress constitutive tyrosine phosphorylation in FLT3-ITD AML [3, 4]. A recent phase III randomized study (RATIFY) demonstrated the survival benefit of a combination of chemotherapy with FLT3 TKIs, leading to the approval of the FLT3 inhibitor midostaurin by the US Food and Drug Administration [5]. However, therapeutic responses to the currently available FLT3 TKIs, if any, are short-lived and followed by early relapse in nearly all cases [4, 6, 7]; accordingly, the development of resistance to these TKIs impedes their therapeutic efficacy. Secondary mutations in the FLT3-TK domain have been demonstrated as one of the mechanisms underlying this resistance [6]. Multiple FLT3-TK domain mutations have been identified in therapy-resistant patients and cell lines [3, 6]. Therefore, the development of inhibitors to block each of these mutations would require a major effort [3, 7]. More recently, mutational analysis of samples from patients who had relapsed after FLT3-TKI treatment, as well as data from preclinical studies suggest that a cellular adaptive mechanism involving the activation of signaling pathways also plays a role in the FLT3-TKI resistance pathway [8], however, these pathways remain poorly elucidated. In addition, the inability of FLT3 TKIs to eliminate leukemia stem cells also contributes to treatment failure. Therefore, novel FLT3-ITD-targeted therapeutic strategies are necessary.

Autophagy is a cell-protective and degradative process that recycles damaged and long-lived cellular components [9]. Autophagy has opposing roles in cancers, depending on the context, oncogenesis, biological features, treatment, and progression [10–12]. In certain cases, autophagy acts primarily as a tumor-suppressive mechanism by maintaining genomic integrity and preventing proliferation and inflammation [12]. Recent evidence suggests that autophagy contributes to cancer initiation and progression, as well as the development of resistance to treatment [10, 12–14]. The dependence of the survival of numerous tumors on autophagy suggests that the inhibition of autophagy represents a therapeutic strategy against cancer that is broadly applicable across various tumor types [15–17]. Autophagy may also have context-specific roles in different types of AML, depending on the clonal origin, oncogenic drivers, and state of leukemia expansion [18]. Autophagy is frequently found to be reduced in human AML blasts, and the loss of key autophagy genes has been shown to lead to leukemia initiation and progression in a mouse model. Disruption of autophagy and downregulation of autophagy-related genes are reportedly involved in the initiation and progression of AML. In contrast, recent studies have revealed that leukemic blasts and leukemia stem cells can utilize autophagy to respond to the specific energy demands during accelerated proliferation, and to counteract chemotherapeutic stress [19, 20]. These mechanisms may be employed to enhance the sensitivity of chemotherapeutic agents targeting leukemia cells. Furthermore, autophagy represents an attractive therapeutic target for various cancers, especially for autophagy-addicted tumors [15–17, 21]. Accordingly, autophagy inactivation by pharmacological inhibition or knockout of essential autophagy-related genes sensitizes specific subsets of AML cells to chemotherapies and small-molecule inhibitors, reduces the proportion of functional leukemia-initiating cells, and extends patient survival [19]. To date, most clinical efforts have focused on the use of general autophagy inhibitors, including chloroquine, hydroxychloroquine, and bafilomycin A1, either as a single agent or in combination with other anti-cancer drugs [14]. However, the development of potent and selective autophagy inhibitors is difficult, in part because most core autophagic proteins used in small-molecule inhibitor screening techniques function far downstream in the pathway and are not readily druggable [22, 23]. A major challenge related to the use of autophagy-targeting drugs is their lack of potent and selective activity [23].

Among the ATG proteins that mediate all steps of autophagy flux, uncoordinated 51-like kinase 1 (ULK1) has serine/threonine protein kinase activity and is considered a key protein in the regulation of autophagy initiation [24, 25]. ULK1 forms a stable complex with ATG13, FIP200, and ATG101 to regulate the recruitment of the VPS34 complex, which consists of VPS34, beclin-1, and ATG14L [25]. In addition, ULK1 acts in later stages of the autophagy pathway, including autophagosome maturation. ULK1 undergoes complex post-translational modifications, including mammalian target of rapamycin complex 1 (mTORC1) and AMP-activated protein kinase (AMPK) [26], depending on the cellular conditions. ULK1 has been shown to promote cell survival in several cancers [27]. Overexpression of ULK1 is negatively correlated with prognosis in various cancers, including colorectal cancer, breast cancer, human nasopharyngeal carcinoma, and esophageal squamous cell carcinoma [28].

ULK1 is considered a promising target for autophagy inhibition because of its central role in pathway activation, druggable nature, and apparent selectivity for autophagy over other cellular functions [29]. Several small-molecule inhibitors of ULK1 have been recently reported. Although a second mammalian ATG1 ortholog, ULK2, also promotes autophagy, the loss of ULK1 alone is sufficient to abrogate autophagy in many cell types, underscoring its particularly important role. The crystal structures of ULK1 have provided insights into the druggable pockets of kinase [30], leading to the development of several inhibitors, including SBI-0206965, MRT 68921, and recently, ULK-101 [27]. Although they are still in the early stages of development, these agents provide new opportunities for the pharmacologic inhibition of autophagy that can potentially be translated to the clinic.

In this study, we evaluated the effects of the ULK1 inhibitors MRT 68921 and SBI-0206965 on apoptosis, and the potential of therapeutic strategies involving ULK1 targeting in FLT3-ITD AML cells. In particular, we aimed to elucidate the mechanisms underlying the effects of these ULK1 inhibitors on FLT3-ITD AML cell death.

## Materials and methods

### Cell lines and culture

The human FLT3-ITD-mutated myeloid leukemia cell lines MV4;11 and MOLM-13 and the FLT3-ITD-negative myeloid leukemia cell lines HL60 and U937, were purchased from the American Type Culture Collection (Rockville, MD, USA). MV4;11 and MOLM-13 cells were grown in RPMI (Invitrogen, Carlsbad, CA, USA) supplemented with 10% fetal bovine serum (FBS). HL60 and U937 cells were cultured in RPMI-1640 (Invitrogen) supplemented with 10% FBS. Cultures were incubated in a 5% CO_2_ humidified incubator at 37 °C.

### Patients and AML cell isolation

This study adhered to the tenets of the Declaration of Helsinki and was approved by the institutional review of board of Severance Hospital (Seoul, Korea). Study participants provided written informed consent, and all patient and healthy donor samples were coded and linked anonymously. Sample identification was made possible by using a code, and the researchers were provided anonymized clinical information of the linked samples. Human leukemia cells were obtained from diagnostic bone-marrow samples (containing at least 70% blasts) of patients with FLT3-ITD-mutated AML prior to receiving chemotherapy at Yonsei University Severance Hospital between 2006 and 2015. For comparative analysis, leukemia cells were also obtained form FLT3-WT AML patients with normal karyotype. Normal, CD34 positive (CD34 (+)) cells were obtained from healthy donors. Mononuclear cells from bone marrow were isolated by Ficoll–Hypaque (Sigma-Aldrich, St. Louis, MO, USA) density gradient centrifugation, and then cryopreserved without being cultured. All methods were carried out in accordance with the relevant guidelines and regulations.

### Reagents

Stock solutions of reagents were prepared by dissolving the reagents in dimethyl sulfoxide (DMSO, Sigma-Aldrich). SBI-0206965, GSK 2606414, and MRT 68921 were purchased from Selleck Chemicals (Houston, TX, USA). Bafilomycin A1 and 3-methyladenine (3-MA) were purchased from Sigma-Aldrich. Hydroxychloroquine (HCQ) was purchased from Myung-in Pharmaceuticals. z-VAD-FMK was obtained from R&D Systems. Control cells were treated with equal amounts of DMSO.

### Establishment of the pMY-puro-FLT3-ITD stable cell line

A suspension of 2 × 10^6^ U937 cells was transfected with pMY-puro-FLT3-ITD (5 μg) using program T-20 of the Amaxa Nucleofector device (Lonza Cologne GmbH, Cologne, Germany) according to the manufacturer’s instructions. Immediately after electroporation, the cells were resuspended in complete medium and incubated at 37 °C in a humidified atmosphere containing 5% CO_2_ for 24 h. pMY-puro-FLT3-ITD-transduced U937 cells were selected on puromycin (2–10 μg/ml) for 2 months.

### Apoptosis analysis

Cells were seeded in 12-well plates at a density of 1 × 10^5^ cells/well. SBI-0206965 and MRT 68921 were added directly to the culture medium at the desired concentration. The final concentration of DMSO did not exceed 0.1% (v/v), which is nontoxic to the cells. After treatment with different doses of the ULK1 inhibitors for the indicated periods, cells were harvested and subjected to annexin V assays on an LSR Fortessa flow cytometer (BD Biosciences). The cells were then resuspended in Annexin V binding buffer, and incubated with Annexin V-FITC (BD Pharmingen) and propidium iodide (PI), and stained with anti-CD34-APC (BD Biosciences) for 30 min for the identification of the CD34+ cell fraction. Data were analyzed using FACSuite software (BD Biosciences).

### Determination of the mitochondrial membrane potential (MMP)

The MMP was monitored using DiOC6, as described previously [31]. We used the DePsipher™ Kit, which uses a unique cationic dye (5,5′6,6′-tetrachloro-1,1′,3,3’tetraethylbenzimidazolylcarbocyanine iodide) to indicate the loss of MMP. For each condition, 1 × 10^6^ cells were incubated with 1 mL of DePsipher™ solution (Trevigen, Gaithersburg, MD, USA) in a 5% CO_2_ incubator at 37 °C for 20 min and then washed with 1 mL of prewarmed 1X reaction buffer with stabilizer solution prior to analysis on a LSR Fortessa flow cytometer (488 nm argon laser). Data were analyzed using FACSuite.

### Transfection of green fluorescent protein (GFP)-microtubule-associated protein 1 light chain 3 (LC3)

A GFP-LC3 construct was generated as described previously [20]. Briefly, a suspension of 2 × 10^6^ leukemia cells was transfected with GFP-LC3 cDNA (5 μg) using program V-01 of the Amaxa Nucleofector 2b device. Immediately after electroporation, the cells were resuspended in complete medium and incubated at 37 °C in a humidified atmosphere containing 5% CO_2_ for 24 h. Cells expressing GFP-tagged LC3 were used to evaluate autophagy induction.

### Confocal microscopy

Cells were centrifuged at 800×*g* onto glass slides, and coverslips were mounted with aqueous mounting medium (Dako) with DAPI (Sigma-Aldrich). Fluorescent signals were analyzed using a Zeiss LSM 700 laser-scanning confocal microscope (Göttingen, Germany). The number of LC3 puncta per cell was quantified as described elsewhere [26]. To estimate the average number of LC3 puncta per cell in each treatment group, 20 cells were randomly selected, and puncta in each cell were manually counted. Results are expressed as the mean of at least three independent experiments.

### Transmission electron microscopy (TEM)

TEM was carried out using standard procedures. Briefly, cells were fixed with 2% glutaraldehyde/paraformaldehyde, pelleted, and treated with 1% osmium tetroxide (Polysciences). The cells were then embedded in pure, fresh resin. Thin sections were double stained with 6% uranyl acetate and lead citrate. The sections were analyzed by TEM (JEM-1011, JEOL, Tokyo, Japan) at 80 kV.

### Western blot analysis

Total cell lysates were prepared and analyzed by western blotting as described previously [32]. The proteins were recovered in sodium dodecyl sulfate (SDS) buffer, separated by SDS-polyacrylamide gel electrophoresis, and transferred onto a nitrocellulose membrane. The membrane was blotted with appropriate primary and secondary antibodies. Rabbit polyclonal antibodies against ULK1, p-ULK1^S757^, FLT3, p-FLT3, STAT5, p-STAT5, p-MEK, p-ERK, caspase-9, caspase-3, PARP, AMPKα, p-AMPKα^T172^, mTOR, p-mTOR^S2448^, p70S6K, p-p70S6K^T389^, PERK, and p-eIF2a were purchased from Cell Signaling Technology (Danvers, MA, USA). Rabbit polyclonal antibodies against LC3 and p-ATG13^S318^ were obtained from Novus Biologicals (Littleton, CO, USA). Mouse anti-p62/SQSTM antibodies were procured from Abnova (Taipei, Taiwan). Anti-p-PERK^T982^ antibodies were purchased from Abcam (Cambridge, UK). Rabbit anti-p-ULK1^S555^ and mouse anti-α-tubulin monoclonal antibodies were obtained from Merck Millipore (Billerica, MA, USA). The secondary antibodies were coupled to horseradish peroxidase. The blots were visualized using an enhanced chemiluminescence (GE Healthcare Bio-Sciences; RPN2232).

### Statistical analysis

Results are expressed as the mean ± standard deviation (SD) of at least three independent experiments. Means of two groups were compared using the two-tailed Student t test. Statistical analysis was performed using GraphPad Prism 4.0 (GraphPad Software Inc). *P* < 0.05 was considered to indicate statistically significant.

## Results

### Effects of ULK1 inhibitors on apoptosis in FLT3-ITD AML

**Fig. 1:**
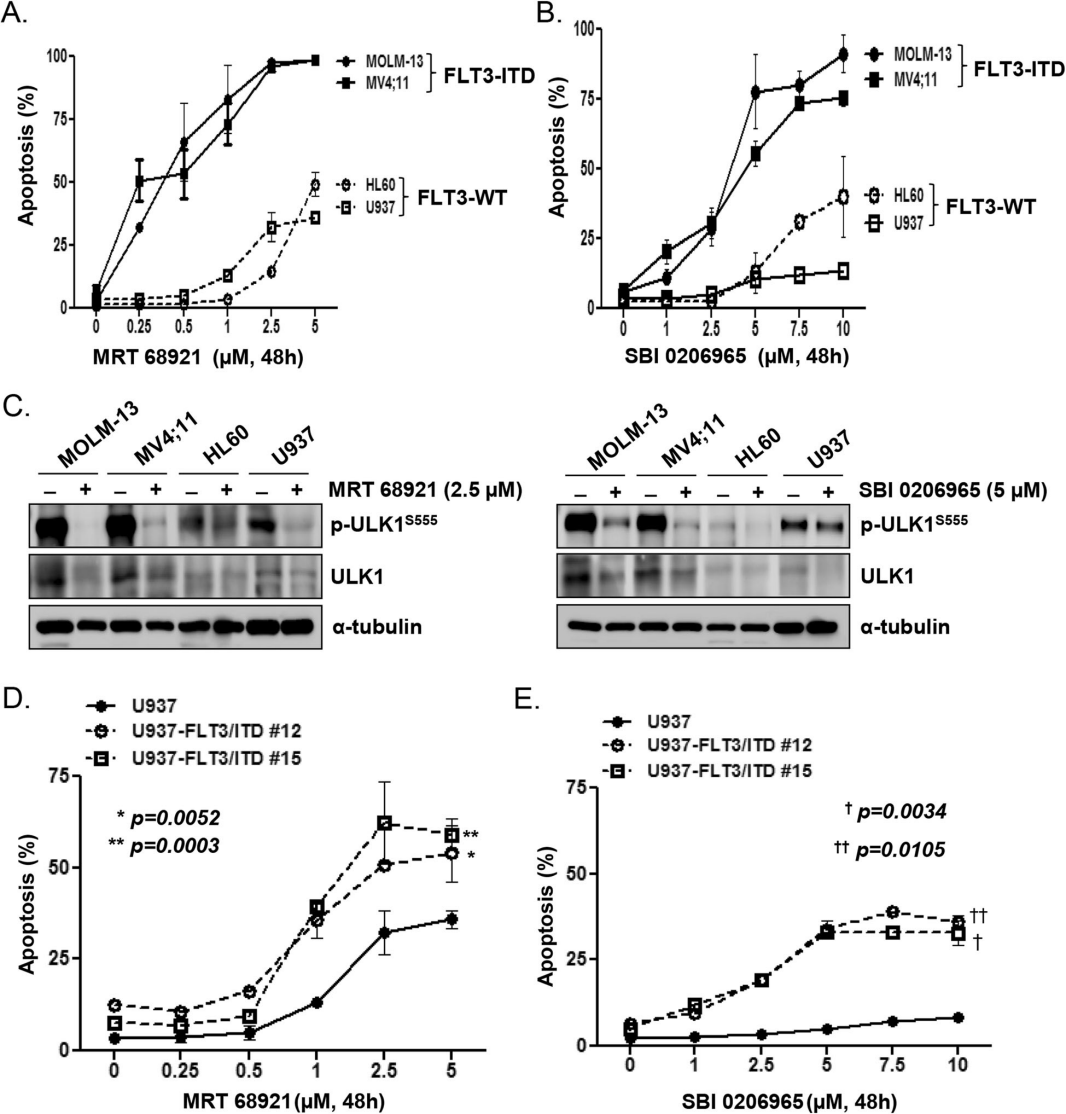
ULK1 inhibition promotes apoptosis in FLT3-ITD AML cell lines. **a**–**e** FLT3-ITD AML cell lines (MOLM-13, MV4;11), FLT3-WT AML cell lines (HL60, U937), and FLT3-ITD-inducible U937 cell lines (U937/FLT3-ITD #12, #15) were treated with various concentration of ULK1 inhibitors (MRT 68921; 2.5 μM, SBI-0206965; 5 μM) for 48 h. **a**, **b** The fraction of apoptotic cells in FLT3-ITD and FLT3-WT AML cell lines treated with the ULK1 inhibitors was analyzed by flow cytometry based on Annexin-V/PI exclusion. **c** Cell lysates were subjected to western blotting for ULK1 expression before and after treatment of the cell lines with the ULK1 inhibitors. α-Tubulin was used as a loading control. **d**, **e** The fraction of apoptotic cells in U937 AML and U937/FLT3-ITD #12, #15 cell lines treated with the ULK1 inhibitors was analyzed by flow cytometry based on Annexin-V/PI exclusion

We assessed the effect of ULK1 inhibitor treatment relative to that of DMSO as a control, on the induction of cell death in a panel of leukemic cell lines harboring FLT3-ITD mutations or not over a 48-h time course. MRT 68921, an inhibitor of ULK1 and ULK2, effectively induced apoptosis in the FLT3-ITD cell lines MV4;11 and MOLM-13 in a dose-dependent manner. However, the extent of apoptosis induced by MRT 68921 in the FLT3-WT cell lines HL-60 and U937 was significantly lower than that in the FLT3-ITD cell lines (Fig. 1a). The ULK1-specific inhibitor SBI-0206965 also induced apoptosis more potently in the FLT3-ITD cell lines than in the FLT3-WT cell lines (Fig. 1b). ULK1 protein levels were increased in FLT3-ITD cell lines compared with those in FLT3-WT cell lines (Fig. 1c). After treatment of the ULK1 inhibitors MRT 68921 and SBI-0206965, phosphorylation of ULK1 was suppressed. Thus, we speculated that ULK1 has a significant role in regulating cell survival in FLT3-ITD AML cells. To confirm that FLT3-ITD mutation was associated with ULK1 inhibitor-induced apoptosis, we established FLT3-ITD-inducible U937 cell lines (U937/FLT3-ITD #12 and #15) and compared ULK1 inhibitor-induced cell death in these cells with that in parental U937 cells (Additional file 1; Fig. S1). The U937/FLT3-ITD cell lines were significantly more sensitive to MRT 68921 (*p =* 0.052; #12 versus parental cells, *p =* 0.0003; #15 versus parental cells) (Fig. 1d) and SBI-0206965 (*p =* 0.0105; #12 versus parental cells, *p =* 0.0034; #15 versus parental cells) (Fig. 1e), indicating that the FLT3-ITD mutation is related to ULK1 inhibitor-induced apoptosis.

### ULK1 inhibitor-induced cell death in primary FLT3-ITD leukemic blasts but not in normal CD34 (+) cells

**Fig. 2:**
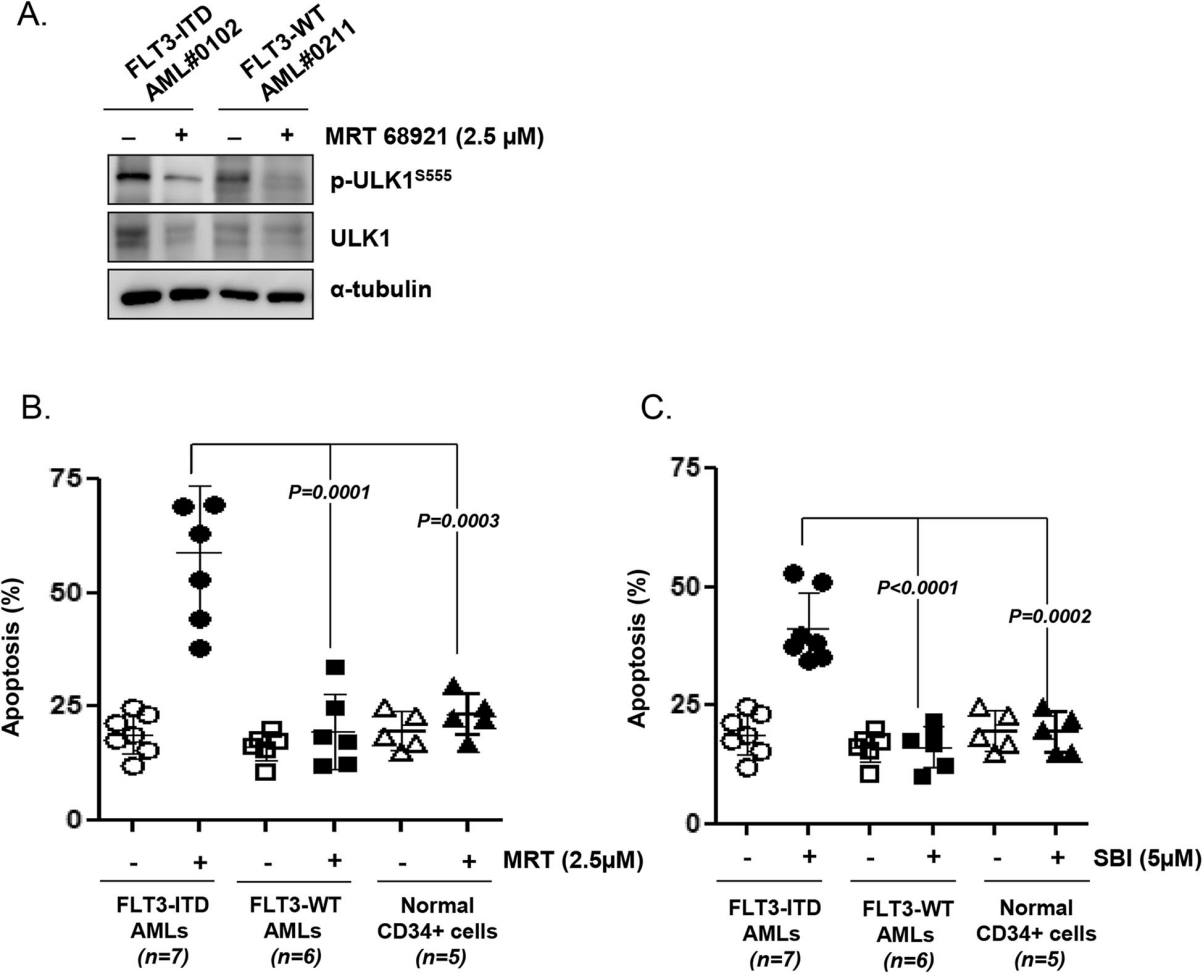
ULK1 inhibition induces selective apoptosis in FLT3-ITD AML patient-derived cells. **a** Cell lysates were subjected to western blotting for ULK1 expression before and after treatment of FLT3-ITD mutated (FLT3-ITD AML #0102) and FLT3-WT AML (FLT3-WT AML #0211) patients cells with the ULK1 inhibitor (MRT 68921; 2.5 μM). α-Tubulin was used as a loading control. **b**, **c** Primary leukemic blasts obtained from FLT3-ITD mutated and FLT3-WT AML patients and normal CD34+ cells obtained from healthy donors were incubated with ULK1 inhibitors (MRT 68921; 2.5 μM, SBI-0206965; 5 μM) for 48 h. The fraction of apoptotic cells in FLT3-ITD AML, FLT3-WT AML patient cells and normal CD34+ cells treated with the ULK1 inhibitors was analyzed by flow cytometry based on Annexin-V/PI exclusion. NS, not significant

Next, we assessed the effects of MRT 68921 and SBI-0206965 on primary leukemic blasts obtained from FLT3-ITD (+) (*n* = 7) and FLT3-WT AML patients (*n* = 6) and normal CD34 (+) cells obtained from healthy donors for allogeneic stem cell transplantation (*n* = 5). As observed in case of the cell line, ULK1 protein levels were increased in FLT3-ITD (+) AML patient cells compared with those in FLT3-WT AML patient cells (Fig. 2a). As shown in Supplementary Table S1 (Additional file 2) and Fig. 2b and c, the extent of MRT 68921- or SBI-0206965-induced apoptosis was significantly higher in the primary FLT3-ITD blasts than in the leukemia cells obtained from FLT3-WT AML cases (MRT 68921; *p =* 0.0001, SBI-0206965; *p <* 0.0001). Normal CD34 (+) cells were relatively unaffected by MRT 68921 (Fig. 2b) or SBI-0206965 (Fig. 2c). These results suggested that the ULK1 inhibitors specifically target FLT3-ITD AML cells, cells, but not normal CD34 (+) cells.

### Caspase activation by ULK1 inhibitors

**Fig. 3:**
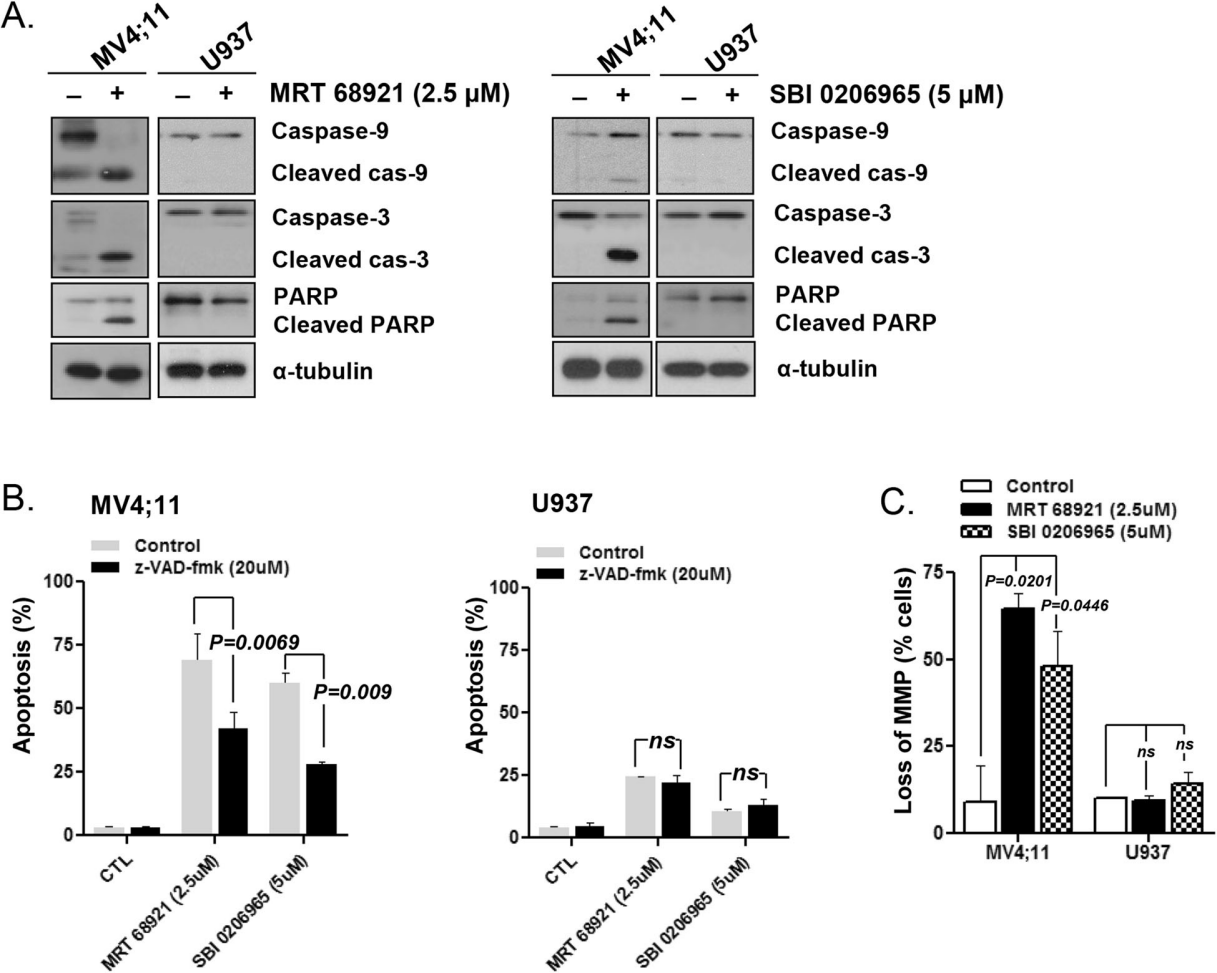
Caspase activity upon ULK1 inhibition in FLT3-ITD AML cells. **a** Cleaved caspase-3 and -9 and PARP levels were examined after treatment of MV4;11 cells and U937 cells with ULK1 inhibitors (MRT 68921; 2.5 μM, SBI-0206965; 5 μM) for 48 h by western blot analysis. α-Tubulin was used as a loading control. **b** After pre-treatment with the pan-caspase inhibitor z-VAD-fmk (20 μM), cells were treated with ULK1 inhibitors (MRT 68921; 2.5 μM, SBI-0206965; 5 μM). The fraction of apoptotic cells in MV4;11 cells and U937 cells was analyzed by flow cytometry based on Annexin-V/PI exclusion. **c** The MMP was quantified by measuring membrane potential-sensitive dye (DiOC6) intensity in MV4;11 and U937 cells after treatment of ULK1 inhibitors (MRT 68921; 2.5 μM, SBI-0206965; 5 μM). CTL, control. NS, not significant

To further analyze the mechanism of ULK1 inhibitor-induced apoptosis further, PARP cleavage by activated caspases was investigated by western blot analysis. As shown in Fig. 3a, MRT 68921 or SBI-0206965 treatment increased the levels of the cleaved caspase-3 and -9, and PARP proteins in MV4;11 cells. Furthermore, pretreatment with the pan-caspase inhibitor z-VAD-fmk (20 μM) for 2 h diminished the MRT 68921- (*p =* 0.0069) and SBI-0206965-induced cell death (*p =* 0.009) of MV4;11 cells (Fig. 3b). These findings indicated that caspase activation may be partly attributed to ULK1 inhibitor-induced apoptosis in FLT3-ITD AML cells. MMP disruption after MRT 68921 or SBI-0206965 treatment was assessed by measuring the mitochondrial uptake of the membrane potential-sensitive dye DiOC6 in MV4;11 and U937 cells. When the cells were treated with 2.5 μM MRT 68921 or 5 μM SBI-0206965 for 48 h, the population of the cell population that lost MMP was 64.73 ± 4.31% and 48.1 ± 10.0%, respectively in MV4;11 cells (Fig. 3c). In contrast, the ULK1 inhibitors did not affect on the mitochondrial membrane potential or apoptosis in U937 cells. This suggested that the apoptosis-inducing mechanism triggered by ULK1 inhibitors operates in the mitochondria in FLT3-ITD cells.

### ULK1 inhibitor activates prosurvival autophagy

**Fig. 4:**
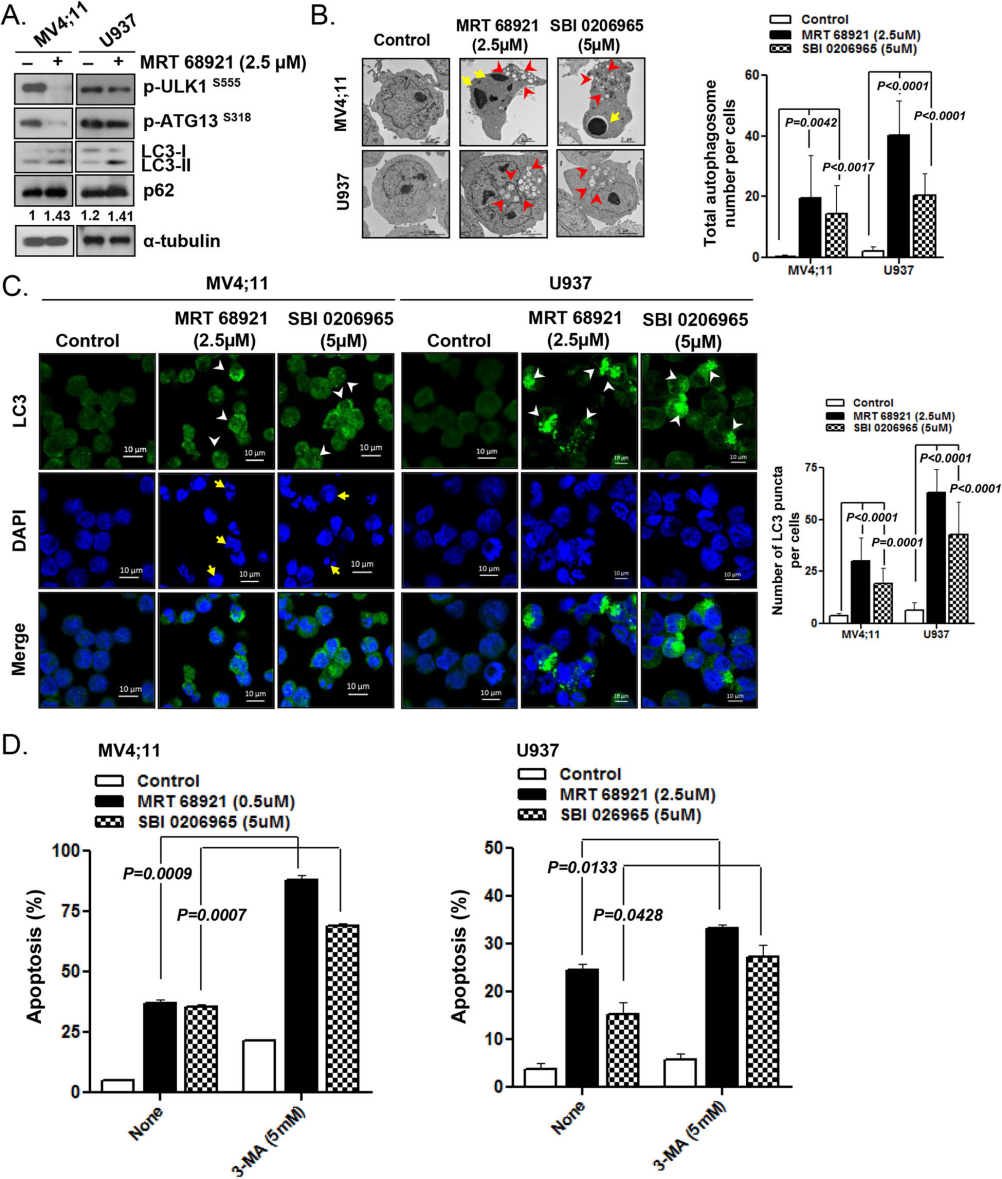
Autophagy induction by ULK1 inhibitors in AML cells. **a** After treatment of MV4;11 cells and U937 cells with 2.5 μM of MRT 68921 for 24 h, cell lysates were subjected to western blotting for p-ULK1, p-ATG13, LC3-I/II and p62. α-Tubulin was used as a loading control. All experiments were performed at least three times independently. **b** TEM-based ultrastructural examination of MV4;11 cells and U937 cells treated with ULK1 inhibitors (MRT 68921; 2.5 μM, SBI-0206965; 5 μM) for 24 h. The arrowheads and arrows indicate autophagosomes and condensed chromatin, respectively. Autophagosomes were counted in at least three different visual fields. Data are the mean ± SD. CTL, control. **c** MV4;11 and U937 cells were treated with ULK1 inhibitors (MRT 68921; 2.5 μM, SBI-0206965; 5 μM) for 24 h and fixed. Cells were stained for LC3B (green) and observed by confocal microscopy. Representative micrographs demonstrate the characteristic punctuate staining, which indicates autophagosome formation. Nuclei were stained with DAPI (blue). The arrowheads indicate LC3 puncta. The arrows denote condensed nuclei. LC3 puncta in each cell were counted in at least three different visual fields. Data are the mean ± SD. **d** MV4;11 and U937 cells were treated with ULK1 inhibitors (MRT 68921; MV4;11, 0.5 μM, U937, 2.5 μM, SBI-0206965; 5 μM) in the presence or absence of autophagy inhibitors 3-MA (5 mM). After incubation for 48 h, the apoptotic fraction was measured by flow cytometry based on Annexin-V/PI exclusion. Data are the mean ± SD

The ULK1 inhibitor is known to inhibit the phosphorylation of ULK1, which regulates autophagy and lysosomal fusion, thereby blocking autophagy flux [33]. To determine whether pharmacologic inhibition of ULK1 affects autophagy pathway in FLT3-ITD AML cells, we evaluated the effects of MRT 68921 on ULK1-downstream molecules p-ATG13, LC3 conversion, and p62 in MV4–11 and U937 cells compared with those in the DMSO control. As shown in Fig. 4a, MRT 68921 induced a decrease in the phosphorylation of ULK1 and ATG13. The level of LC3-II increased upon MRT 68921 treatment in MV4–11 and U937 cells, indicating that autophagy was induced. As expected, p62 accumulated in MRT 68921-treated MV4;11 and U937 cells, suggesting that the ULK1 inhibitor blocked the p62 degradation process rather than activating autophagic flux. Following treatment with MRT 68921 or SBI-0206965, TEM revealed an increased number of autophagosomes in MV4–11 and U937 cells; furthermore, apoptosomes were observed in MV4;11 cells (Fig. 4b). MRT 68921 and SBI-0206965 treatment led to significant increases (greater than 10-fold) in the number of GFP-LC3 puncta in MV4;11 cells (MRT 68921; *p <* 0.0001, SBI-0206965; *p =* 0.0001); even higher increases were observed in U937 cells (MRT 68921; *p* < 0.0001, SBI-0206965; *p <* 0.0001) (Fig. 4c). These results suggest that ULK1 inhibitors induced autophagy in parallel with inhibiting autophagic flux in AML cells. We next examined the functional roles of autophagy induced by ULK1 inhibitors in the AML cell lines. We examined the effects of 3-MA, a type III PI3K inhibitor that suppresses the early steps of autophagy, on MRT 68921- or SBI-0206965-induced apoptosis in MV4;11 and U937 cells. As shown in Fig. 4d, the fraction of apoptotic cells was significantly increased following 3-MA addition in MV4;11 cells (MRT 68921; *p* = 0.0009, SBI-0206965; *p =* 0.0007). These findings indicated that MRT 68921-induced autophagy was mostly prosurvival.

### ULK1 inhibition affects FLT3 signaling

**Fig. 5:**
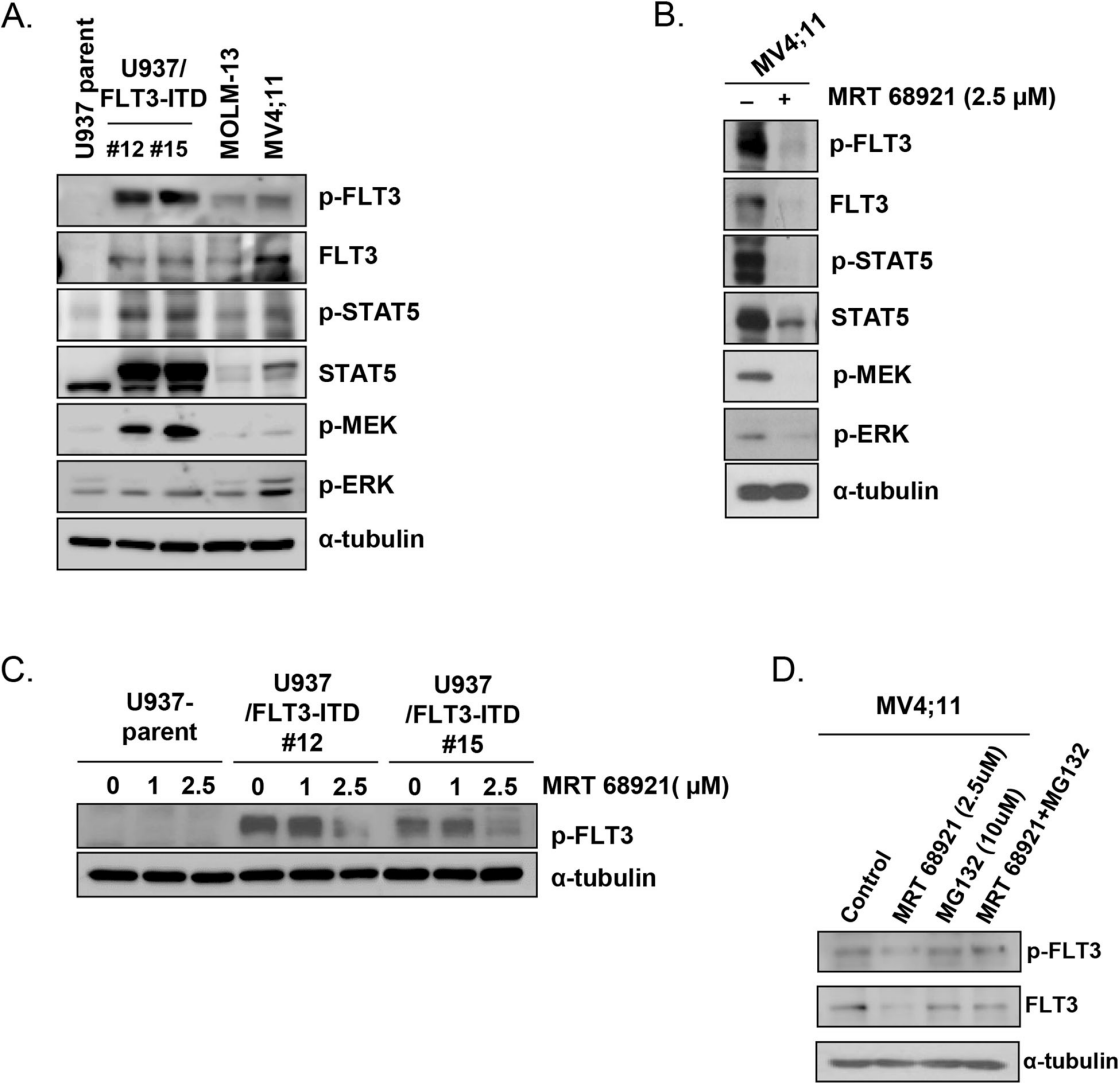
ULK1 inhibition induces FLT3 degradation in FLT3-ITD AML cells. **a** FLT3-ITD AML cell lines (MOLM-13, MV4;11), U937 cells, and FLT3-ITD-inducible U937 cell lines (U937/FLT3-ITD #12, #15) were incubated, and cell lysates were subjected to western blotting using antibodies against FLT3-downstream molecules (FLT3, p-FLT3, STAT5, p-STAT5, p-MEK, p-ERK). α-Tubulin was used as a loading control. **b** MV4;11 cells were incubated with 2.5 μM of MRT 68921 for 48 h, and cell lysates were subjected to western blotting using antibodies against the FLT3-downstream molecules. α-Tubulin was used as a loading control. **c** Cell lysates were subjected to western blotting for FLT3 expression before and after treatment of FLT3-ITD-inducible U937 cell lines (U937/FLT3-ITD #12, #15) with the ULK1 inhibitor (MRT 68921; 1, 2.5 μM). α-Tubulin was used as a loading control. **d** MV4;11 cell lysates were subjected to western blotting for FLT3 expression after treatment of the cells with ULK1 inhibitor (MRT 68921; 2.5 μM), proteasome inhibitor (MG132; 10 μM), or both for 48 h

To investigate why ULK1 inhibition potentially targets FLT3-ITD AML cells, we assessed its effect on the FLT3 signaling pathway. MV4–11, MOLM-13, and FLT3-ITD-inducible U937 cells exhibited FLT3 phosphorylation and activation of the downstream signaling cascade, as evidenced by STAT5, MEK, and ERK phosphorylation (Fig. 5a). Interestingly, MRT 68921 attenuated FLT3 protein expression and suppressed FLT3, STAT5, MEK, and ERK phosphorylation in MV4–11 cells (Fig. 5b). The increased FLT3 phosphorylation levels in the U937/FLT3-ITD cell lines was also suppressed by MRT 68921 in a dose-dependent manner (Fig. 5c). Next, we investigated the mechanism underlying MRT 68921-induced FLT3 downregulation in MV4–11 cells. Addition of the proteasome inhibitor MG132 to MRT 68921 delayed the decrease in FLT3, indicating that ULK1 inhibition accelerated proteasomal degradation of FLT3 (Fig. 5d). Confocal microscopy revealed that LC3-positive structures and FLT3 molecules were present at different locations within the cell after MRT 68921 treatment (Additional file 1; Fig. S2A), indicating that FLT3 was not degraded through MRT 68921-induced autophagy. Inhibition of autophagy with 3-MA increased MRT 68921-induced FLT3 degradation (Additional file 1; Fig. S2B). These results indicate that in FLT3-ITD AML cells, MRT 68921 reduced FLT3 expression at the protein level, and autophagy played a role in protecting cells from this effect.

### Unfolded protein response (UPR) under ULK1 inhibition

We observed the effects of MRT 68921 on the UPR by western blotting. MRT 68921 suppressed the phosphorylation of PERK and eIF2α in MV4;11 cells,, but increased the phosphorylated PERK and eIF2α levels in U937 cells (Additional file 1: Fig. S1A). Phosphorylation of PERK and eIF2α in MV4;11 cells was augmented in a dose-dependent manner upon treatment with the PERK-specific activator tunicamycin (Additional file 1: Fig. S1B). However, pretreatment with tunicamycin did not abrogate MRT 68921-induced apoptosis in MV4–11 cells (Additional file 1: Fig. S1C). Suppression of PERK with the PERK-specific inhibitor GKS 2606414 did not affect MRT 68921-induced apoptosis in U937 cells (Additional file 1: Fig. S1D). Thus, MRT 68921 treatment increased apoptosis in FLT3-ITD AML cells, regardless of PERK signaling activation.

## Discussion

Increasing evidence suggests that dysregulated autophagy is a hallmark of malignant disorders and plays complex roles in the tumorigenesis and progression of cancers and their resistance to treatment, in a context-dependent manner [10, 11, 31]. The diverse functions of numerous autophagy-related proteins in cancer have been extensively evaluated [31, 34]. Among them, ULK1 has drawn the most interest because it plays a key role in autophagy initiation as an only serine/threonine protein kinase [35, 36]. The findings that high levels of ULK1 are associated with poor prognosis in several solid tumors [28, 37] and that ULK1 inhibition can rescue the phenotype induced by caspase-3 deletion in AML1/ETO AML cells [38] suggest that ULK1 is a promising new therapeutic target for cancers.

FLT3-ITD mutations activate the survival pathway in an oncogene-addicted state. Given the critical role of autophagy in the cell biology of FLT3-ITD AML [39], ULK1 is likely to play an important role in this AML subtype. The higher levels of activated ULK1 and its downstream molecules in FLT3-ITD cells observed in our study suggest that ULK1 is indispensable for survival in FLT3-ITD-mutated cells and that its selective inhibition may potentially induce cell death. We demonstrated here for the first time that two ULK1 inhibitors, MRT 68921 and SBI-0206965, induced caspase-dependent apoptosis preferentially in FLT3-ITD-mutated leukemia cell lines and primary leukemic blasts obtained from FLT3-ITD AML patients, while sparing normal CD34 (+) cells.

MRT 68921-mediated apoptosis in FLT3-ITD cells was associated with dose-dependent increases in the cleavage of caspase-3 and -9, and PARP levels. Pretreatment of MV4–11 cells with the pan-caspase inhibitor z-VAD-FMK notably reduced the levels of cleaved caspases and PARP, indicating that ULK1 inhibitors induced apoptosis in a caspase-dependent manner. The interaction between ULK1 and caspase-3 was demonstrated in AML1/ETO leukemia cells [38]. However, the molecular interplay between ULK1 and activation of the caspase cascade in FLT3-ITD AML remains to be elucidated. We found that the ULK1 inhibitor increased apoptosis by MMP loss with FLT3 degradation in FLT3/ITDAML cells. Further research is needed to clarify the mechanism by which FLT3-ITD prevents mitochondrial apoptosis. Ju, H. Q et al. showed that FLT3-ITD causes an increase in aerobic glycolysis through AKT-mediated upregulation of mitochondrial hexokinase, which regulates the mitochondrial permeability transition pore [40]. Recently, studies in various FLT3ITD AML models provided evidence that FLT3 inhibition induces mitochondrial oxidative stress that determines the triggering of an apoptotic response [41, 42]. Our study suggests that the role of ULK1 in mitochondrial membrane potential and mechanisms by which the ULK1 inhibitor induces mitochondrial apoptosis in FLT3/ITD cells requires further analysis.

ULK1 inhibition also induced cell death, albeit to a lesser degree, in FLT3-WT leukemia cells, necessitating a clarification of whether the apoptosis-inducing effects of ULK1 inhibition were primarily due to ULK1 inhibition or off-target effects thereof. Both MRT 68921 and SBI-0206965 inhibited ULK1 activity, as demonstrated by the reduction in phosphorylation of ULK1 downstream molecules ATG13 (Ser318) [43], beclin-1 (Ser14) [25], and VPS34 (Ser249) [44] as well as ULK1 itself (T180) [24]. Phosphorylation of ATG13 at serine 318 is directly correlated with ULK1 activity [45]. We observed that the extent of apoptosis was highly correlated with the reduction in ATG13 phosphorylation in FLT3-ITD cells.

Although MRT68921 and SBI026965 were developed to inhibit autophagy [29, 46], the action of these ULK1 inhibitors remains unclear. Particularly, the action spectrum of these ULK1 inhibitors has not been fully evaluated in cancer cells, including leukemia blasts, which are considered to have abnormal autophagy regulatory mechanism in a context-dependent manner. In addition, most published data described the autophagy inhibitory effects of the ULK1 inhibitors in the settings of induced autophagy, not in the basal conditions. Interestingly, both MRT 68921 and SBI-0206965 activated LC3-II conversion and GFP-LC3 puncta and autophagosome formation, which are indicators of autophagy induction. However, these results did not confirm that autophagic flux was increased. The increase in p62 level after ULK1 inhibitor treatment indicated that autophagic flux was blocked by the ULK1 inhibitor. These results suggest that the ULK1 inhibitors induced autophagic flux blockage in parallel with autophagy activation. Gross autophagy revealed offset results for both actions. Although the ULK1 inhibitor suppressed p62 degradation, the accumulation of LC3 II, autophagosomes, and LC3 puncta were lower in MV4;11 cells than in U937 cells, indicating that autophagic flux is greater in MV4;11 cells. We demonstrated that this activated autophagy in FLT3/ITD AML cells played a prosurvival role by enhancing ULK1 inhibitor-induced apoptosis using the autophagy inhibitor. Reagents conventionally used for autophagy inhibition have different mechanisms and action targets. 3-MA inhibits autophagy by blocking autophagosome formation, whereas bafilomycin A1 and HCQ inhibit the late phase of autophagy. Since the ULK1 inhibitor activated autophagosome formation, which is the early phase of autophagy, the apoptosis-inducing effects of an addition of 3-MA, rather than BafA1 or HCQ, on ULK1 inhibitors were investigated.

The mechanisms by which ULK1 inhibiors result in the activation of prosurvival autophagy in FLT3-ITD cells remain to be investigated. Under stress conditions, AMPK can inactivate mTORC1 by phosphorylating the mTOR upstream regulator TSC2 at the Thr1227 and Ser1354 residues, leading to autophagy activation [47]. The feedback regulatory mechanism between AMPK and ULK1 remains to be further elucidated. Cheong H et al. showed that ammonia production due to amino acid catabolism is responsible for ULK1-independent autophagy [48]. A recent study suggested that the requirement for ULK1 in autophagy induction can be bypassed by an increase in VPS34 kinase activity, caused by inhibition of a newly identified and negative regulatory VPS34 acetylation event [49]. Additional analysis of ULK1-independent pathways operating in autophagy may support our findings.

The direct molecular interactions between ULK1 and the FLT3-ITD-mutated protein are unknown. In this study, treatment of MV4–11 cells with ULK1 inhibitors led to decreases in the levels of phospho-FLT3, as well as p-ERK and p-STAT5, which are associated with FLT3 activation. These findings indicate that ULK1 inhibition led to the suppression of FLT3 signaling. It has been shown that autophagy induces the degradation of oncogenic fusion proteins in AML, leading to cell differentiation and death of leukemic blasts [50, 51]. Furthermore, the FLT3-ITD-mutated protein is post-translationally regulated and degraded by autophagy [39]. However, in this study, we found that FLT3-ITD molecules and autophagosomes are located at different sites in MV4;11 cells treated with ULK1 inhibitors. This indicates that ULK1 inhibition-induced autophagy may not be the mechanism underlying FLT3-ITD degradation. The addition of the autophagy inhibitor 3-MA to MRT 68921 led to decreases in the levels of p-FLT3; therefore, autophagy was speculated that play a protective role against FLT3 degradation in FLT3-ITD AML cells. In addition, proteasome inhibition partially attenuated MRT 68921-induced apoptosis, suggesting that proteasomal degradation of FLT3-ITD contributed to the ULK1 inhibition-mediated reduction in the levels of activated FLT3 and its downstream molecules.

The UPR, which involves three major transducers, PERK, IRE1, and ATF6, occurs in response to intracellular and extracellular events that perturb protein folding in the endoplasmic reticulum (ER) [52]. Autophagy and UPR pathways are interconnected and regulate cellular responses to apoptotic triggers such as environmental and genetic stresses in cancer cells [12, 52]. ER stress (ERS) is thought to activate autophagy via UPR-mediated transcriptional upregulation of the autophagic machinery [53]. We evaluated changes in the activation of the PERK/eIF2α/ATF4 pathway, which is one of the essential branches of the UPR, after treatment of MV4–11 cells with the ULK1 inhibitors. PERK, eIF2α, and ATF4 phosphorylation decreased upon treatment with MRT 68921, indicating that the ERS pathway is suppressed following ULK1 inhibition. However, when AML cells were treated with MRT 68921 plus PERK activator or inhibitor, there were no significant changes in apoptosis This indicates that ERS does not play a critical role in the ULK1 inhibition-mediated regulation of apoptosis and autophagy in FLT3-ITD cells.

## Conclusions

To our knowledge, the present study is the first to demonstrate that ULK1 inhibitors effectively induce mitochondria-mediated, caspase-dependent apoptosis in FLT3-ITD-mutated leukemia cell lines and primary leukemia cells, but not in normal CD34 (+) cells. Paradoxically, ULK1 inhibitors also induced prosurvival autophagy, as evidenced by the synergistic enhancement of apoptosis following the addition of autophagy inhibitors. Further studies are necessary to determine whether ULK1 inhibition is an effective strategy for overcoming FLT3-TKI resistance. Owing to its central role in autophagy regulation, druggable nature, and apparent selectivity over other autophagy inhibitors, along with minimal effect on normal CD34 (+) cells, ULK1 represents a promising molecular target for FLT3-ITD AML treatment.

## Supplementary information

**Additional file 1: Figure S1.** FLT3-ITD expression in FLT3-ITD-inducible U937 cell lines. Cell lysates were subjected to western blotting for FLT3-ITD expression of FLT3-ITD-inducible U937 cell lines (U937/FLT3-ITD #10, #11, #12, #13, #14, #15). GAPDH was used as a loading control. **Figure S2**. The effects of 3-MA on ULK1 inhibitor-induced FLT3 degradation in FLT3-ITD AML cells. (A) MV4;11 cells were treated with 2.5 μM of MRT 68921 for 24 h and fixed. Cells were stained for LC3B (green) and FLT3 (red) and observed by confocal microscopy. Nuclei were stained with DAPI. (B) MV4;11 cell lysates were subjected to western blotting for FLT3 expression after treatment of the cells with ULK1 inhibitor (MRT 68921; 2.5 μM), autophagy inhibitor (3-MA; 5 mM), or both for 48 h **Figure S3**. Unfolded protein response and ULK1 inhibition in FLT3-ITD AML cells. (A) MV4;11 and U937 cells were incubated with 2.5 μM of MRT 68921 for 24 h, and cell lysates were subjected to western blotting using antibodies against the indicated molecules (PERK, p-PERK, p-elF2a). α-Tubulin was used as a loading control. (B) MV4;11 cells were incubated with various concentrations of the PERK activator tunicamycin for 48 h. (C) The fraction of apoptotic cells in MV4;11 cells treated with MRT 68921 (0.5, 1, or 2.5 μM) in the presence or absence of tunicamycin (0.1 or 0.125 μg/ml) was analyzed by flow cytometry based on Annexin-V/PI exclusion. (D) The fraction of apoptotic cells in U937 cells treated with ULK1 inhibitors (MRT 68921; 2.5 μM, SBI-0206965; 5 μM) in the presence or absence of the PERK inhibitor GSK 2606414 (20 μM) was analyzed by flow cytometry based on Annexin-V/PI exclusion. **Figure S4.** Densitometry analyses on the entire western blot experiments. (A-C) **Figure S5.** Densitometry analyses on the entire western blot experiments. (D-H)

**Additional file 2: Table S1**. Effects of ULK1 inhibitors on phenotypes and apoptosis of primary acute myeloid leukemia FLT3 cells

## Data Availability

The datasets used and/or analysed during the current study are available from the corresponding author on reasonable request.

## Abbreviations

FLT3-ITD: FMS-like kinase 3 internal tandem duplication
AML: Acute myeloid leukemia
TKIs: Tyrosine kinase inhibitors
ULK1: Uncoordinated 51-like kinase 1
FBS: Fetal Bovine Serum
CD34 (+): CD34 positive
mTORC1: Mammalian target of rapamycin complex 1
AMPK: AMP-activated protein kinase
3-MA: 3-methyladenine
HCQ: Hydroxchloroquine
PI: Propium iodide
MPP: Mitochondrial membrane potential
SDS: Sodium dodecyl sulfate
UPR: Unfolded protein response
ER: Endoplasmic reticulum
ERS: ER stress
SD: Standard deviation
