# Supplementary figures and images for: ULK1 inhibition as a targeted therapeutic strategy for FLT3-ITD-mutated acute myeloid leukemia
Source: J Exp Clin Cancer Res. 2020 May 11;39:85. doi: 10.1186/s13046-020-01580-4 (PMC7212592; doi:10.1186/s13046-020-01580-4)

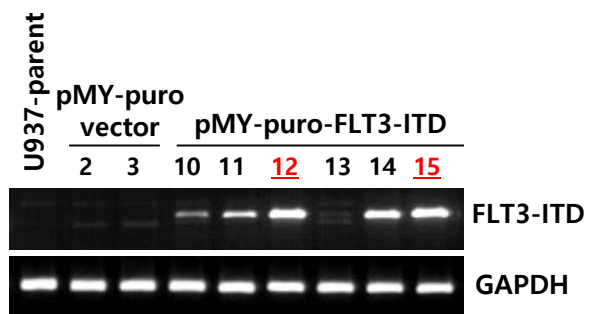

**Figure S1.**

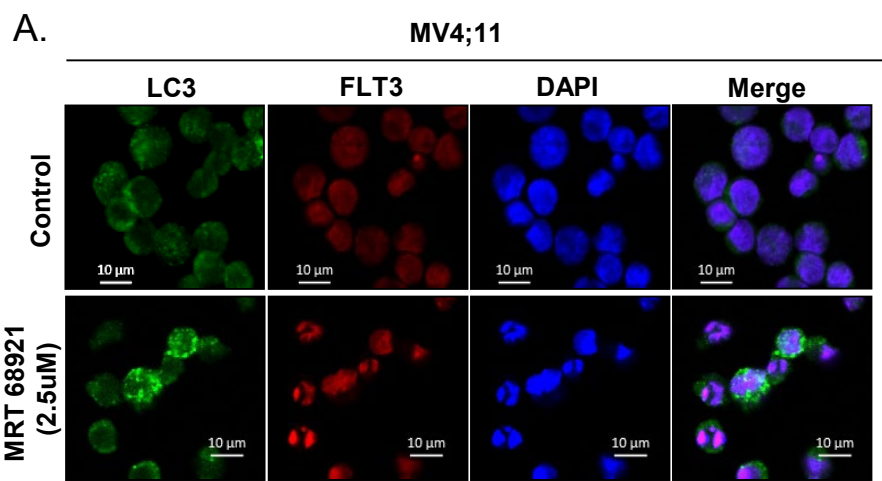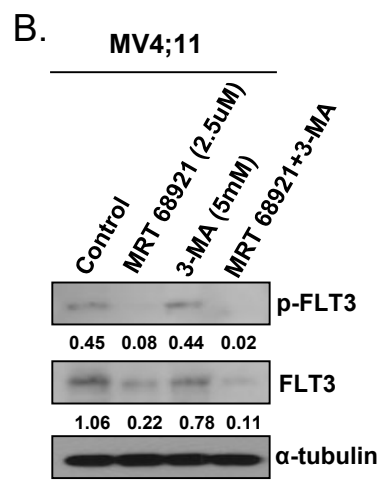

Figure S2.

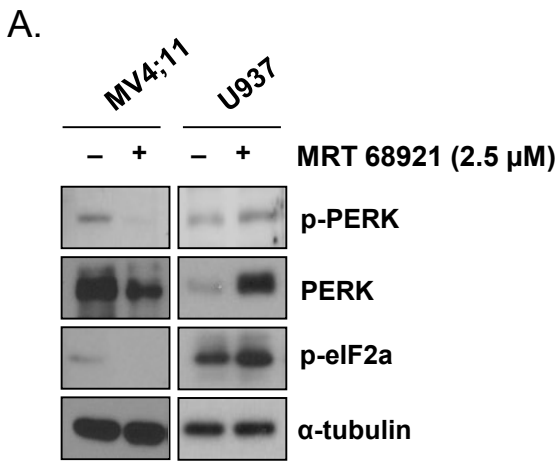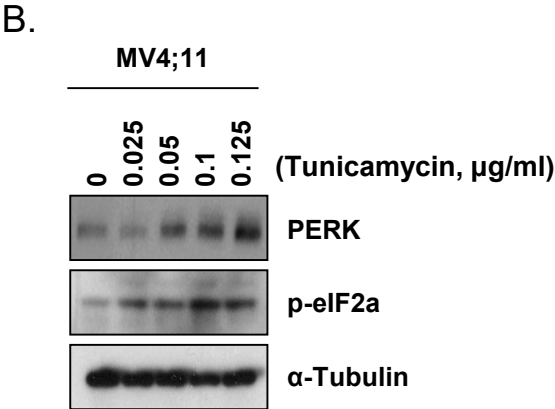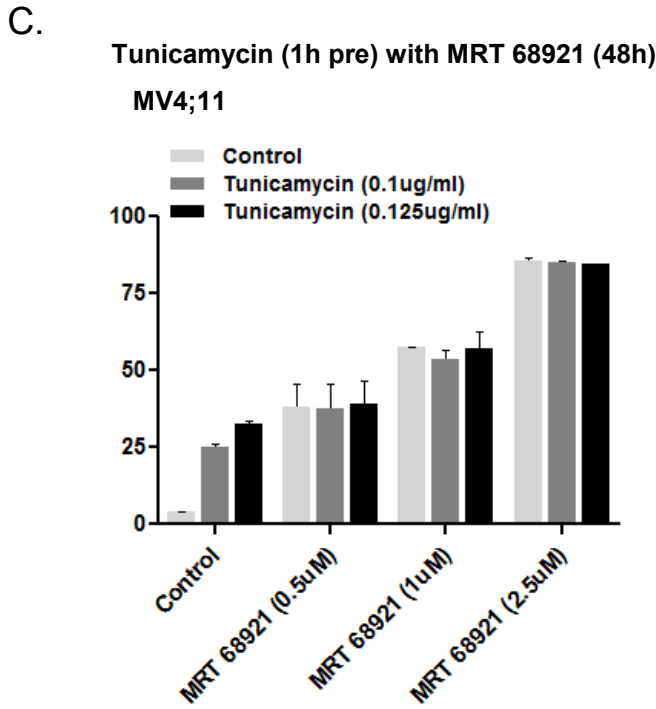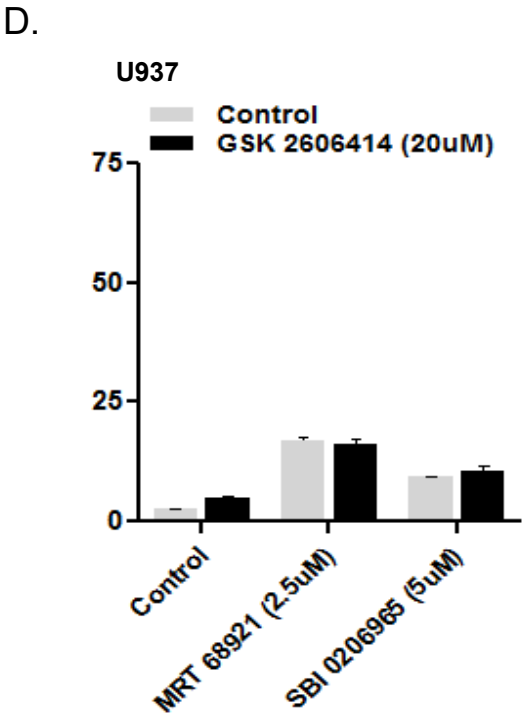

Figure S3.

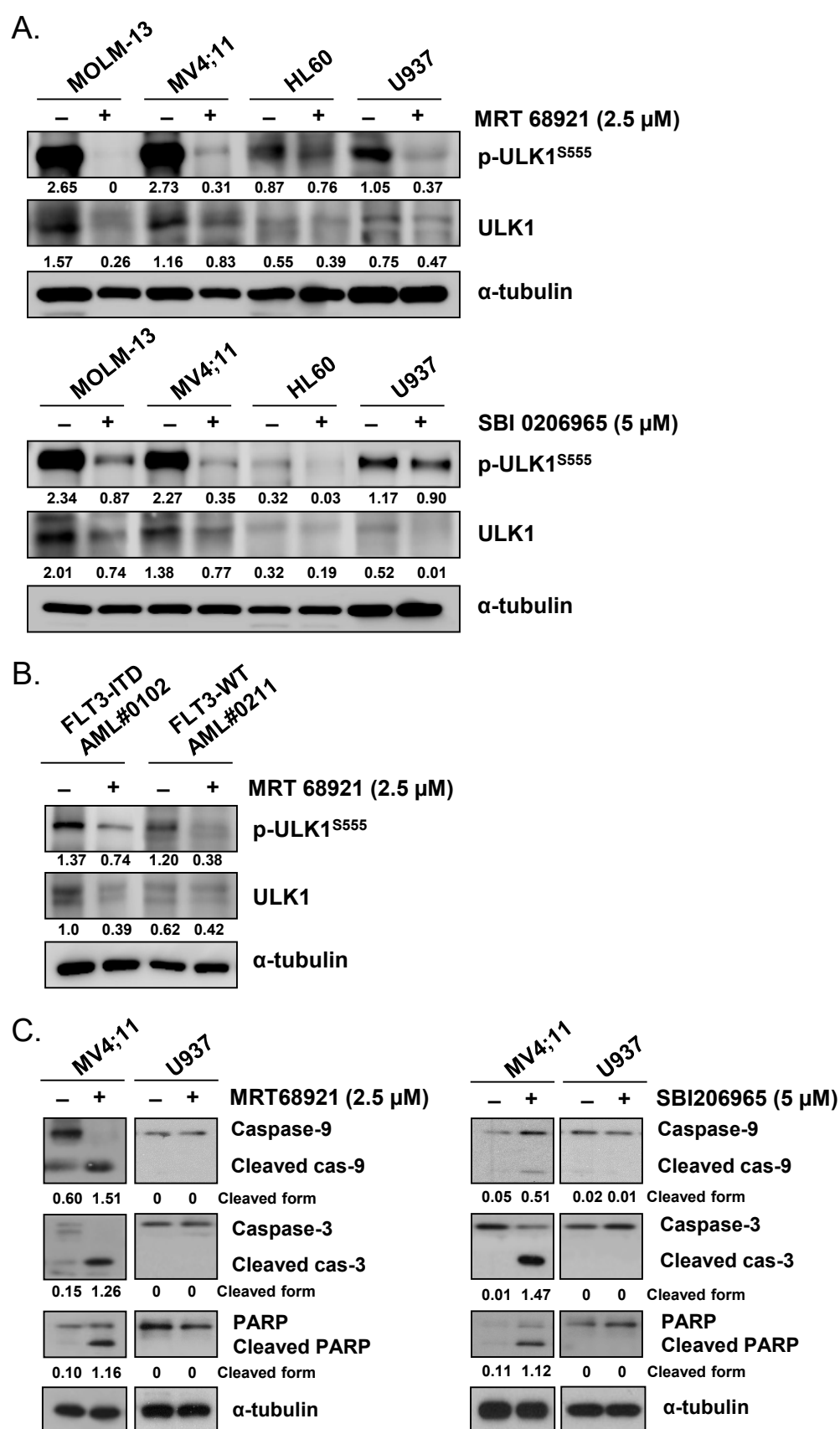

Figure S4.

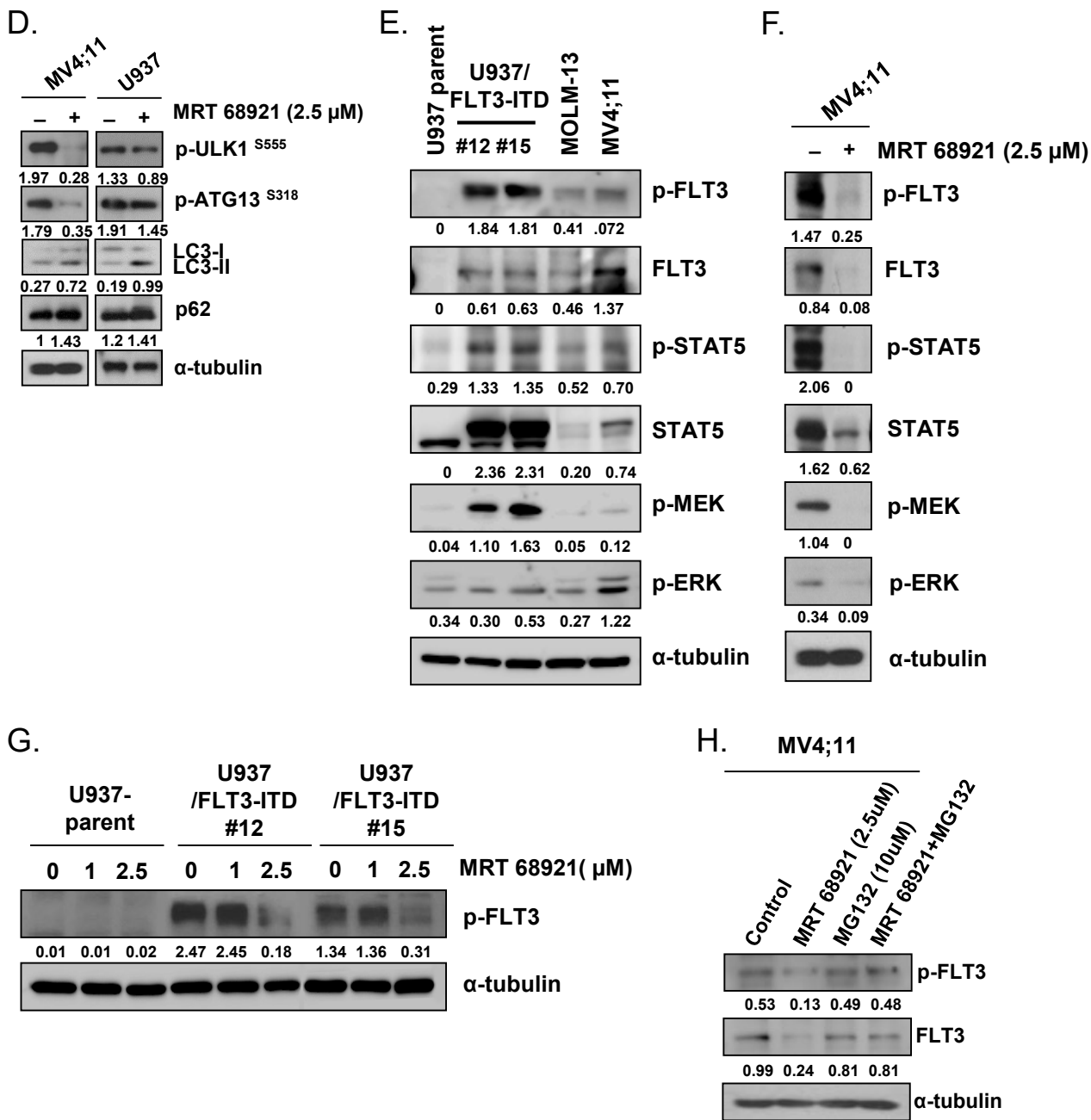

Figure S5.

Supplement: Supplementary file 1 — Additional file 1: Figure S1. FLT3-ITD expression in FLT3-ITD-inducible U937 cell lines. Cell lysates were subjected to western blotting for FLT3-ITD expression of FLT3-ITD-inducible U937 cell lines (U937/FLT3-ITD #10, #11, #12, #13, #14, #15). GAPDH was used as a loading control. Figure S2. The effects of 3-MA on ULK1 inhibitor-induced FLT3 degradation in FLT3-ITD AML cells. (A) MV4;11 cells were treated with 2.5 μM of MRT 68921 for 24 h and fixed. Cells were stained for LC3B (green) and FLT3 (red) and observed by confocal microscopy. Nuclei were stained with DAPI. (B) MV4;11 cell lysates were subjected to western blotting for FLT3 expression after treatment of the cells with ULK1 inhibitor (MRT 68921; 2.5 μM), autophagy inhibitor (3-MA; 5 mM), or both for 48 h Figure S3. Unfolded protein response and ULK1 inhibition in FLT3-ITD AML cells. (A) MV4;11 and U937 cells were incubated with 2.5 μM of MRT 68921 for 24 h, and cell lysates were subjected to western blotting using antibodies against the indicated molecules (PERK, p-PERK, p-elF2a). α-Tubulin was used as a loading control. (B) MV4;11 cells were incubated with various concentrations of the PERK activator tunicamycin for 48 h. (C) The fraction of apoptotic cells in MV4;11 cells treated with MRT 68921 (0.5, 1, or 2.5 μM) in the presence or absence of tunicamycin (0.1 or 0.125 μg/ml) was analyzed by flow cytometry based on Annexin-V/PI exclusion. (D) The fraction of apoptotic cells in U937 cells treated with ULK1 inhibitors (MRT 68921; 2.5 μM, SBI-0206965; 5 μM) in the presence or absence of the PERK inhibitor GSK 2606414 (20 μM) was analyzed by flow cytometry based on Annexin-V/PI exclusion. Figure S4. Densitometry analyses on the entire western blot experiments. (A-C) Figure S5. Densitometry analyses on the entire western blot experiments. (D-H) [file 13046_2020_1580_MOESM1_ESM.pdf]
