# Supplementary material for: ULK1 inhibition as a targeted therapeutic strategy for FLT3-ITD-mutated acute myeloid leukemia
Source: J Exp Clin Cancer Res. 2020 May 11;39:85. doi: 10.1186/s13046-020-01580-4 (PMC7212592; doi:10.1186/s13046-020-01580-4)
Supplement: Supplementary file 2 — Additional file 2: Table S1. Effects of ULK1 inhibitors on phenotypes and apoptosis of primary acute myeloid leukemia FLT3 cells [file 13046_2020_1580_MOESM2_ESM.docx]

**Table S1. Effects of ULK1 inhibitors on phenotypes and apoptosis of primary acute myeloid leukemia FLT3 cells**

| **Patient No. #** | **Sex/Age** | **Chromosome** | **Mutation** | **Source** | **MRT 68921 (2.5µM, 48 h)** | **SBI 0206965 (10µM, 48 h)** |
| --- | --- | --- | --- | --- | --- | --- |
|  |  |  | ***FLT3-ITD*** |  | **AnnexinV^+^ PI^+^, %** | **Annexin V^+^ PI^+^, %** |
| YH0102 | F/45 | 46, XX | POS | BM | 63.11 | 38.25 |
| YH0105 | M/37 | 46, XY | POS | BM | 37.80 | 37.64 |
| YH0111 | F/34 | 46, XX | POS | BM | 44.40 | 39.82 |
| YH0112 | M/65 | 46, XY | POS | BM | 52.84 | 35.15 |
| YH0115 | M/39 | 46, XY | POS | BM | 76.92 | 51.01 |
| YH0120 | M/65 | 46, XX | POS | BM | 69.14 | 34.71 |
| YH0122 | M/25 | 46, XY | POS | BM | 69.37 | 52.97 |
| YH0201 | M/23 | 46, XY | NEG | BM | 11.90 | 10.14 |
| YH0202 | M/48 | 46, XX | NEG | BM | 18.41 | 17.71 |
| YH0205 | M/78 | 46, XY | NEG | BM | 17.20 | 21.97 |
| YH0207 | F/55 | 46, XX | NEG | BM | 12.47 | 12.43 |
| YH0211 | F/63 | 46, XX | NEG | BM | 33.73 | 18.83 |
| YH0219 | M/56 | 46, XY | NEG | BM | 24.63 | 17.06 |
| Donor01 | M/25 | 46, XX | NEG | PBSC | 24.77 | 22.16 |
| Donor02 | F/18 | 46, XY | NEG | PBSC | 22.51 | 19.75 |
| Donor03 | F/70 | 46, XY | NEG | PBSC | 22.09 | 15.18 |
| Donor04 | F/37 | 46, XY | NEG | PBSC | 17.12 | 14.96 |
| Donor05 | M/67 | 46, XY | NEG | PBSC | 29.66 | 24.86 |

*Note.* POS, positive; NEG, negative; BM, bone marrow; PBSC, peripheral blood stem cell
